# Examining healthcare transition experiences among youth living with HIV in Atlanta, Georgia, USA: a longitudinal qualitative study

**DOI:** 10.1002/jia2.25676

**Published:** 2021-02-22

**Authors:** Alexis S Halyard, Kamini Doraivelu, Andrés F Camacho‐González, Carlos del Río, Sophia A Hussen

**Affiliations:** ^1^ Emory University School of Medicine Atlanta GA USA; ^2^ Hubert Department of Global Health Emory University Rollins School of Public Health Atlanta GA USA; ^3^ Division of Infectious Diseases Department of Pediatrics Emory University School of Medicine Atlanta GA USA; ^4^ Division of Infectious Diseases Department of Medicine Emory University School of Medicine Atlanta GA USA

**Keywords:** adolescent, youth, HIV, healthcare transition, care engagement

## Abstract

**Introduction:**

Virtually all youth living with HIV in paediatric/adolescent care must eventually transition to adult‐oriented HIV care settings. To date, there is limited evidence examining the perspectives of youth living with HIV longitudinally through the healthcare transition process. The objective of our study was to examine attitudes and experiences of youth living with HIV regarding healthcare transition, including potential change in attitudes and experiences over time.

**Methods:**

We conducted a longitudinal qualitative interview study within a large, comprehensive HIV care centre in Atlanta, Georgia, USA between August 2016 and October 2019.We interviewed 28 youth living with HIV as part of a longitudinal observational cohort study of youth undergoing healthcare transition. We conducted qualitative interviews both immediately prior to, and one year following the transition from paediatric to adult‐oriented care.

**Results:**

Six distinct themes emerged from interviews conducted with youth living with HIV pre‐transition: (1) reluctance to transition; (2) paediatric spaces as welcoming, and adult spaces as unwelcoming; (3) varying levels of preparation for transition; and (4) expectation of autonomy in the adult clinic. Analysis of post‐transition interviews with the same youth demonstrated: (1) inconsistencies in the transition experience; (2) fear and anxiety about transition quelled by experience; (3) varying reactions to newfound autonomy and (4) communication as the most valuable facilitator of successful transition.

**Conclusions:**

This study’s longitudinal perspective on the healthcare transition experience yields insights that can be incorporated into programming targeting this critically important population. Although our study was conducted in a USA‐based clinic with co‐located paediatric and adult care services, many of our findings are likely to have relevance in other settings as well. Interventions aiming to improve HIV care engagement through transition should seek to enhance patient–provider communication in both paediatric and adult clinics, improve preparation of patients in paediatric clinics and ease patients gradually into autonomous disease management.

## 1 INTRODUCTION

Healthcare transition from paediatric to adult‐oriented care is a high‐risk time for disengagement among youth living with HIV [1, 2, 3]. Paediatric and adult‐oriented healthcare settings have been described as two different medical sub‐cultures [4]. In paediatric settings, the model of care tends to be interdisciplinary, with social workers, psychologists and other support staff available to help youth cope with health‐related stressors. In contrast, adult‐oriented care settings typically place more responsibility on patients for their own care, and are less likely to have the resources to offer the same level and intensity of support. These cultural differences between clinics may have significant clinical consequences: a study analyzing HIV outcomes in a multicentre USA‐based cohort found that youth living with HIV receiving care in adult medical settings were more likely to discontinue antiretroviral therapy when compared to those receiving care in paediatric settings [5]. Similarly, a South African study found that youth had higher retention rates if they remained in paediatric care, when compared with a historical cohort that was transitioned to adult care [6]. These disparities highlight the importance of careful planning and follow‐up when transitioning youth living with HIV from paediatric to adult care settings.

Healthcare transition is defined as “the purposeful, planned movement of adolescents and young adults with chronic physical and medical conditions from child‐centred to adult‐oriented healthcare systems” [7]. When executed effectively, healthcare transition can empower patients to gain autonomy and become stewards of their own health. Conversely, poorly executed transition may lead to care disengagement and adverse downstream health consequences including the development of resistant viral strains, declining immune status and increased potential for HIV transmission [2, 8]. Unfortunately, suboptimal transition experiences are far from uncommon. A multi‐site prospective study of youth living with HIV in the USA found that only 37% of transition‐eligible youth were linked to adult care within a 9‐month follow‐up period [9], and prior retrospective studies based in the USA, Thailand and the Netherlands have documented care retention rates ranging from 50% to 89% one year after transition [3, 10, 11, 12, 13]. There is therefore a critical need for evidence‐based interventions to keep youth living with HIV engaged in care before, during and after transition. An important first step towards developing such interventions is improving our understanding of experiences of youth living with HIV throughout the transition process – a goal that is particularly well‐suited to qualitative research.

A number of qualitative studies, mostly based in the USA, have examined attitudes of youth living with HIV prior to healthcare transition. Most studies in this area focus exclusively on youth who acquired HIV vertically (e.g. perinatally) as opposed to youth who acquired HIV horizontally (e.g. those infected in adolescence, primarily through sexual contact). This line of research primarily highlights the reluctance of youth living with HIV to undergo transition, often due to close relationships with healthcare providers who have cared for them for many years [14, 15]. Fewer studies have examined attitudes post‐transition [16, 17]. This work highlights differences between care settings, but also invokes transition as a time associated with opportunities for self‐improvement. We sought to build on this literature by examining experiences before and after healthcare transition within a longitudinal cohort of youth living with HIV in the USA.

## 2 METHODS

We conducted a prospective, observational mixed‐methods cohort study of healthcare transition among youth at a large HIV care centre in Atlanta, Georgia, USA. This centre contains both a paediatric and an adult‐oriented clinic within the same building; they are in physically separate locations (i.e. different floors) and most providers work exclusively in one section or the other. Patients are typically transitioned from the paediatric to the adult‐oriented clinic around the time of their 25th birthday. To date, the clinic lacks a formal transition protocol to guide this process, and we have previously found evidence of transition‐related disruption to care in this setting [12, 18].

We recruited 70 participants from the paediatric clinic between August 2016 and June 2018, within three months prior to anticipated healthcare transition. Patients were approached for participation if they were approaching their 25th birthday within the next three months, and were eligible as long as they were willing and able to provide consent. We followed the cohort with serial surveys and medical chart abstractions over the subsequent year, in order to determine clinical care outcomes and to examine their experiences through this process. We selected a subset of 28 participants for a qualitative sub‐study consisting of two in‐depth interviews. We selected qualitative participants based on participant availability/interest and a purposive sampling strategy, making sure to include women and youth with vertically acquired infection to ensure that these minority viewpoints were represented.

Each qualitative sub‐study participant was asked to participate in two interviews: A baseline (pre‐transition) interview conducted between the last paediatric and first adult clinic visits, and a follow‐up (post‐transition) interview conducted approximately one year later. Interviewers used a semi‐structured guide to elicit information about the participants’ healthcare experiences. Most interviews were conducted in a private room within the clinic; some follow‐up interviews were conducted via phone for participant convenience. Participants received $50 USD upon completion of each interview.

### 2.1 Ethical statement

All participants provided written informed consent prior to engaging in any study activities. The study was approved by the Emory Institutional Review Board and Grady Research Oversight Committee. Qualitative data collection occurred between December 2016 and October 2019.

### 2.2 Data analysis

Interviews were recorded and transcribed verbatim, then imported into MAXQDA 18 qualitative software (Berlin, Germany) for coding and thematic analysis [19]. We utilized a team coding approach to enhance reliability and internal validity. First, deductive codes from the interview guide were applied to the transcripts to highlight themes and guide comparisons. Next, inductive codes representing emerging themes were added to the transcripts. Two study team members independently coded transcripts, and then reconvened to discuss and resolve discrepancies. After inter‐coder reliability was established, the remaining transcripts were coded independently. Analysts then wrote memos describing coding categories, emerging themes and relationships between constructs and codes. We also compared the frequency and content of coded text between different participant sub‐groups (e.g. youth who were horizontally vs. vertically infected; men vs. women).

## 3 RESULTS

### 3.1 Pre‐transition interviews

The mean age of participants was 24 years. Most identified as Black/African‐American (92.9%), male (75%), gay (60.7%) and acquired HIV horizontally (75%; see Table 1 for summary of demographics and Table 2 for individual participant characteristics). Themes emerging from the baseline interviews included: (1) reluctance to transition; (2) paediatric spaces as more welcoming than adult spaces; (3) varying preparation for transition and (4) expectation of autonomy in the adult clinic.

**Table 1:** Participant demographics

Mean age (years)	24.4 ± 0.5
Gender: n (%)	
Cisgender male	21 (75.0)
Cisgender female	7 (25.0)
Race: n (%)	
Black/African‐American	26 (92.9)
Other	2 (7.2)
Sexual orientation: n (%)	
Heterosexual/straight	8 (28.6)
Gay, bisexual or questioning	19 (67.9)
Mode of transmission: n (%)	
Vertical	7 (25.0)
Horizontal	21 (75.0)
Education level: n (%)	
Less than high school	2 (7.1)
High school diploma or GED	5 (17.9)
Some tech school/college	16 (57.1)
College graduate	4 (14.3)
Employment status	
Not currently employed	5 (17.9)
Part‐time	10 (35.7)
Full‐time	12 (42.9)
Annual income	
<$10,000	11 (39.3)
$10,000‐$19,999	7 (25.0)
$20,000‐$29,999	6 (21.4)
$30,000‐$49,999	3 (10.7)

**Table 2:** Individual participant characteristics

Participant number	Mode of acquisition	Gender	Completed post‐transition interview	Transition description at 12 months
1	Horizontal	Male	Yes	Engaged in adult care^a^
2	Horizontal	Male	Yes	Engaged in adult care (outside of our system)
3	Vertical	Male	Yes	Engaged in adult care (outside of our system)
4	Horizontal	Male	Yes	Engaged in adult care
5	Horizontal	Male	Yes	Engaged in adult care (outside of our system)
6	Horizontal	Male	Yes	Engaged in adult care
7	Horizontal	Male	Yes	Linked^b^ but disengaged from adult care
8	Horizontal	Male	Yes	Disengaged, not linked to adult care
9	Horizontal	Male	Yes	Engaged in adult care
10	Vertical	Female	Yes	Engaged in adult care
11	Horizontal	Male	Yes	Engaged in adult care
12	Horizontal	Male	Yes	Not linked to adult care, moved outside of our system
13	Horizontal	Male	Yes	Engaged in adult care
14	Horizontal	Male	No	Disengaged, not linked to adult care
15	Horizontal	Male	Yes	Engaged in adult care
16	Horizontal	Male	Yes	Engaged in adult care
17	Horizontal	Male	No	Engaged in adult care
18	Horizontal	Male	Yes	Engaged in adult care
19	Horizontal	Male	Yes	Engaged in adult care
20	Horizontal	Male	No	Engaged in adult care
21	Horizontal	Female	Yes	Linked but disengaged from adult care
22	Vertical	Male	Yes	Still engaged in pediatric care
23	Vertical	Female	Yes	Linked but disengaged from adult care
24	Vertical	Female	Yes	Engaged in adult care
25	Vertical	Female	Yes	Engaged in adult care
26	Vertical	Female	Yes	Still in pediatric care
27	Horizontal	Female	Yes	Engaged in adult care
28	Horizontal	Male	No	Engaged in adult care

^b^ engaged in adult care: seen at least twice in the 12‐month period

^c^ linked to adult care: refers to attending one appointment with the adult care provider.

#### 3.1.1 Reluctance to transition

Reluctance to transition was nearly universal. This sentiment was pervasive regardless of mode of acquisition; though youth who were infected vertically related this reluctance to their experiences receiving care at the paediatric clinic since infancy. Sadness over losing the relationship with paediatric providers was the main contributor to reluctance, as below:

I don’t like it, I don’t want to [transition]. I don’t know, it was told to me, but I don’t remember. I just know that I don’t want to do it. I am not even trying to remember. Because I don’t want to leave [provider name]. (Participant 21, female, horizontally acquired)

Conversely, if a participant lacked a close patient–provider relationship, their attitude towards transition was often more ambivalent:

I don’t see the same doctor every time I come. So seeing another different person is like “okay!”…I’m a lab rat! I see different people, but that’s like… it’s fine. I’m fine with seeing different people. (Participant 27, female, horizontally acquired)

Participants also valued relationships with other paediatric staff, particularly social service providers. Paediatric staff helped youth remain engaged in care, and participants appreciated this assistance – adding to their reluctance to leave.

[Social service provider name]…she'll just reach out, hey, [participant name], how you doing? Just making sure I'm good. Just are you having any problems, is everything okay? Have you been able to book your appointment? If I miss my appointment, I can email her and say, 'I missed my appointment, can you help?' 'Oh, let me help you out.'… that's why I love the pediatric. That's why I don't want to leave. (Participant 9, male, horizontally acquired)

#### 3.1.2 Paediatric spaces as more welcoming than adult spaces

Participants overwhelmingly appeared to think positively of paediatric clinic spaces (described as “perky,” “happy” and “jolly”) and reflected fondly on their experiences there. This positive atmosphere was enhanced by warm relationships with staff, as described by Participant 1 (male, horizontally acquired): “*Everybody was really welcoming. You know, I felt you could feel the love in the building…They really wanted to, they treated you like family.”* This participant and others described ways in which lack of support from families of origin – often in reaction to sexuality or HIV status – made them appreciate the supportiveness of the paediatric clinic even more: “*I’m used to [Pediatrics] now. [They] make me feel special. And it’s like when you don’t get, get that, that feeling of being special at home, it’s nice to have somebody make you feel special.”*

In contrast, pre‐transition views of adult clinic were mostly negative, and adult‐oriented spaces were seen as unwelcoming. One participant described experiences he had with the adult clinic below:

I wish that some of the people [in adult clinic] were more friendly. They’re not as patient…I mean, you can be personable without hand‐holding. And I feel that they need that. Like, there have been some rude people and it’s been hard to deal with…if I was over the age of Pediatric and I came in [to adult clinic]… and I dealt with some of what I’ve experienced here, I might turn around and feel like well I ain’t gonna get treated. (Participant 15, male, horizontally acquired)

Some participants expressed negative, stigmatizing views about patients receiving care in the adult clinic and accompanying anxiety about sharing a waiting room and clinic space with these patients.

Up here [in pediatric clinic] you see some shit, but down there[in adult clinic] that's like, like the jungle…it's just so many people in and out, like drug addicts, just like, I don't know, it's just a lot. I think I'm just going to go to [name of private clinic]. (Participant 12, male, horizontally‐acquired)

#### 3.1.3 Preparation for transition

Participants described having been alerted about the prospect of transition to varying degrees, by medical or social service providers. Some youth had been introduced to their prospective adult provider and/or received a tour of the adult clinic, but others were still completely unsure of what to expect:

Where do I go, how does it work, who I’m [going to] see, where I have to sit, what’s the process like? [In pediatrics]…I know where the waiting room is, I know who works down here… I kind of know some familiar faces, I know I can talk to my case manager… [In adult clinic] I don’t know what room is what, who’s who. (Participant 18, male, horizontally acquired)

The degree to which patients were oriented to transition appeared to be largely left to the discretion of the individual paediatric provider, leading to substantially different experiences across the cohort.

#### 3.1.4 Expectation of autonomy in adult clinic

A commonly held expectation among youth was that the transition would lead to increased personal responsibility for their health.

I was definitely told that, you know, I just have to be really, really on top of everything, like for myself, you know. Just like how you said it's more of a business type of thing, like no one is really going to hold your hand. (Participant 8, male, horizontally‐acquired)

Although some of the subtext (e.g. lamenting the absence of “handholding” in the adult setting), suggests apprehension towards autonomy, explicit mention of emotions associated with autonomy was absent. Additionally, it remains unclear in what ways that youth were equipped (or not) with the tools to take on their new healthcare role.

### 3.2 Post‐transition interviews

Seventy‐five percent (N = 21) of participants were successfully engaged in adult care after 12 months, and 24 completed follow‐up interviews (Table 2). The following themes emerged from post‐transition interviews: (1) inconsistencies in the transition experience; (2) fear/anxiety about transition quelled by experience; (3) varying reactions to newfound autonomy and (4) communication as the most important facilitator of successful transition.

#### 3.2.1 Inconsistencies in the transition experience

Post‐transition, participants continued to describe highly variable experiences. While some were introduced to their future providers and toured through the adult facilities, others had no substantive orientation to adult clinic spaces prior to their first appointment. This finding echoed discussions of preparation in the pre‐transition interviews; showing that the prior variability was not simply due to discussions or preparation that had yet to occur. Participant 28 (male, horizontally acquired) lamented his lack of preparation: “[The transition] was a bit abrupt, the change, because they did tell me it was going to happen but it just kind of happened one day when I came into my appointment.”

This type of experience, where participants were vaguely aware of impending transition but still found the change abrupt, was common. In contrast, others described having been guided through transition:

[My social worker] explained the process and got me started. He helped me understand what was going on, and knowing that I wasn’t going to have the same doctor because I was going to the adult floor. (Participant 7, male, horizontally‐acquired)

#### 3.2.2 Fear/anxiety about transition quelled by experience

Although pre‐transition interviews showed that patients were often fearful or anxious of healthcare transition, most participants appeared to acclimate to the change by the time of their follow‐up interviews.

I was thinking I’m not going to be able to keep up with my appointments, with my medicine. It had changed, because I find out that it’s the same as the pediatric to adult, like keeping my appointments and my medicine…I was worried about that more than anything. But since I kept my appointments and medicine, I’m kind of calm now. (Participant 6, male, horizontally acquired)

Many participants did not perceive major differences between the two clinic areas. A few stated that they now found the adult clinic more welcoming than they had previously perceived it to be, and many expressed appreciation for their new medical providers as well.

#### 3.2.3 Varying reactions to newfound autonomy

Attitudes towards transition varied depending on whether or not participants valued their own healthcare autonomy. Some participants appreciated a newfound feeling of increased independence in the adult clinic.

I mean, I like all the “Are you okay?” and all the babying and pacifying stuff. But for me that’s not what I need because I’m a person that gets the job done and keep on moving. I like to go down there [to adult clinic], tell me what I need, what I need to do and get out the door. (Participant 4, male, horizontally acquired)

Other participants missed having more active involvement of clinic staff in their care. For them, lack of involvement by the providers contributed to a lack of connection to the adult clinic.

[Adult providers] won't tell you that. It's like it's your job. Up here [in the pediatric clinic], they are on top of it and they will tell you, like, hey, you might want to consider this, you might want to do that…I've heard some people say like, they spoon feed you or something like that. I've never, ever had that happen to me or from what I've seen. I just think that it's a process and I think that's just how the doctor should be. (Participant 5, male, horizontally acquired)

#### 3.2.4 Communication as the most important facilitator of successful transition

Overall, most participants were satisfied with the transition process. However, several participants asked for more support, specifically wishing that they had heard a better explanation of what the process would entail, as described by Participant 11 (male, horizontally‐acquired), who wished he had “*a little bit more of a heads‐up.”* Participants expressed a desire to become better acquainted with their adult‐oriented providers prior to transition, and to also wean the support from the paediatric clinic gradually (e.g. asking to not simultaneously “lose” their paediatric physician and social worker).

They [adult provider] could probably come in to my last two or three appointments in peds and then I’d be like, hey, this is so‐and‐so, this will be your new doctor or provider or whatever the case might be, and then we can build that rapport just starting from there. Then it will be like cool [snaps fingers] a smooth, seamless transition. (Participant 13, male, horizontally acquired)

Overall, participants desired clearer communication with both pre‐transition and post‐transition providers throughout the process – even if they were currently at a point where they felt reasonably acclimated to adult clinic.

### 3.3 Between‐group comparisons

We examined the data to determine whether differences emerged with regards to perceptions or experiences of transition among different groups of youth. We did not elicit different themes expressed, or differences in patterns of expression, by youth based on mode of acquisition, gender or sexual orientation.

## 4 DISCUSSION

We found that youth living with HIV transitioning from paediatric to adult‐oriented care are often anxious about the process, and desire greater communication and consistency from both pre‐ and post‐transition healthcare providers. Our participants described reluctance to transition to adult‐oriented care and a favourable attitude towards paediatric clinic spaces and staff, similar to what has been found in other pre‐transition studies [14, 16]. We built on these findings by following patients after transition to find out how they experienced the transition, and how their attitudes evolved. In spite of participants’ initial critiques, it was reassuring that many anxieties resolved once participants actually experienced care in the adult clinic, and some even appreciated the increased autonomy.

The longitudinal exploration of pre‐ and post‐transition attitudes, lend itself to the subsequent identification of potential points for intervention. Pre‐transition, our results suggest that youth would prefer a more gradual preparatory process including anticipatory guidance about upcoming changes and introductions to new adult clinic providers. Other studies, including those based in the USA and in lower and middle‐income countries (LMICs), have similarly emphasized the importance of a gradual, planned transition and preparation for independent disease management prior to transition [20, 21, 22]. Post‐transition, many participants wanted to be gradually eased into their newfound autonomy – perhaps retaining some contact with paediatric providers and/or support staff even after the first appointment in the adult clinic has been completed. Adult‐oriented providers might also benefit from greater sensitivity to the magnitude of change that patients are experiencing. Our findings here will be relayed to clinic leadership, and are also being incorporated into ongoing intervention‐focused research.

One strength of our study was the intentional inclusion of youth who with both horizontally and vertically acquired HIV, as well as men and women. Different characteristics and challenges have been associated with each population [8], and providers may perceive adolescents as having different needs relating to sexual/reproductive health and psychosocial functioning based on their mode of acquisition [23]. We did not find major differences between demographic groups in terms of transition needs and experiences, and attitudes toward transition seemed to depend less on identity and more on personal experiences with the healthcare system. At the same time, although we tried to represent a range of youth experiences, almost all participants were Black, and most were young sexual minority men (reflecting our local and national epidemic). The healthcare experiences of ethnic and sexual minorities are influenced by structural racism, other institutional biases and medical mistrust. Future work, perhaps in demographically different locations, could seek to specifically compare transition experiences among different racial groups in order to examine the roles of racism and intersecting stigmas in the transition process. Similarly, we did not enrol any youth of transgender experience in our study, and more information about healthcare transition amidst multiple other life transitions in this population would be valuable as well.

### 4.1 Limitations

Our study was conducted in a single centre in the USA, where transition occurs between departments within a single building. Although this might seem an idealized situation that would limit generalizability to other clinical or country settings, our participants experienced many of the same anxieties and observed the same inter‐clinic cultural differences found in other locations. We also had four participants who we were unable to schedule for follow‐up interviews; it is possible that these patients had different transition experiences than those who we successfully interviewed twice. It is also important to note that our participants may not have been completely representative of the clinic population. Youth who disengaged from care prior to transition were not included, as we recruited those attending appointments in the paediatric clinic. Additionally, youth who agreed to participate may be different in some ways from those who did not.

## 5 CONCLUSIONS

This study provides insights into the dynamic nature of healthcare transition, and suggests areas for improvement. Interventions aiming to improve care engagement through transition should seek to enhance patient–provider communication in both paediatric and adult clinics, improve preparation of patients in paediatric clinics and ease patients gradually into autonomous disease management in adult clinics. Future research to develop and test such interventions is urgently needed to improve health and well‐being for youth living with HIV.

## COMPETING INTERESTS

The authors declare no conflicts of interest.

## AUTHORS’ CONTRIBUTIONS

ASH led the drafting of the manuscript, conducted data analysis and contributed to data collection. KD contributed to drafting of the manuscript, participant recruitment and data collection and data analysis. AFC contributed to the conceptualization of the overall study, interpretation of findings and gave feedback/revisions on the final manuscript. CDR contributed to the conceptualization of the overall study, interpretation of findings and gave feedback/revisions on the final manuscript. SAH led conceptualization of the parent study and contributed to drafting of the manuscript, analysis and interpretation of findings.

## ABBREVIATIONS

HIV, Human Immunodeficiency Virus.
